# Study protocol: ASCRIBED: the impact of Acute SystematiC inflammation upon cerebRospinal fluId and blood BiomarkErs of brain inflammation and injury in dementia: a study in acute hip fracture patients

**DOI:** 10.1186/s12883-019-1447-7

**Published:** 2019-09-07

**Authors:** Nick Leavey, Simon P. Hammond, Lee Shepstone, Jane Cross, Henrik Zetterberg, Colm Cunningham, Alasdair MacLullich, Leiv Otto Watne, Anne Marie Minihane, Clive Ballard, Anne-Brita Knapskog, Roanna Hall, Gregory Howard, Matt Hammond, Chris Fox

**Affiliations:** 1 Norwich Clinical Trial Unit, Norwich Medical School, Faculty of Medicine and Health Sciences, University of East Anglia, Norwich Research Park, Norwich, NR4 7TJ England; 2 School of Education and Lifelong Learning, Faculty of Social Sciences, University of East Anglia, Norwich Research Park, Norwich, NR4 7TJ England; 3 Norwich Medical School, Faculty of Medicine and Health Sciences, University of East Anglia, Norwich Research Park, Norwich, NR4 7TJ England; 4 School of Health Sciences, Faculty of Medicine and Health Sciences, University of East Anglia, Norwich Research Park, Norwich, NR4 7TJ England; 5 Department of Psychiatry and Neurochemistry, the Sahlgrenska Academy at the University of Gothenburg, S-431 80 Mölndal, Sweden; 6 Clinical Neurochemistry Laboratory, Sahlgrenska University Hospital, S-431 80 Mölndal, Sweden; 7 Department of Neurodegenerative Disease, UCL Institute of Neurology, Queen Square, London, WC1N 3BG UK; 8 UK Dementia Research Institute at UCL, Cruciform Building, Gower Street, London, WC1E 6BT UK; 9 School of Biochemistry and Immunology, Trinity Biomedical Sciences Institute & Trinity College Institute of Neuroscience, Trinity College Dublin, Dublin, Ireland; 10 Edinburgh Delirium Rese arch Group, Geriatric Medicine, University of Edinburgh, Royal Infirmary Edinburgh, Room S1642, Edinburgh, EH16 4SA UK; 11 Oslo Delirium Research Group, Department of Geriatric Medicine, Oslo University Hospital, PO box 4950, Nydalen, N-0424, Oslo, Norway; 12 Norwich Medical School, BCRE, James Watson Road, University of East Anglia, Norwich, NR4 7UQ England; 13 Wolfson Centre for Age Related Diseases, King’s College London Guy’s Campus, Hodgkin Building, London, SE1 1UL England; 14 Department of Geriatric Medicine, Oslo University Hospital, Ullevaal, Nydalen, Postbox 4956, Oslo, Norway; 15 Department of Geriatric Medicine, University of Edinburgh Room S1642, New Royal Infirmary of Edinburgh, 51 Little France Crescent, Edinburgh, Midlothian, EH16 4SA UK; 16 Department of Psychological Sciences, Norwich Medical School, Faculty of Medicine and Health Sciences, University of East Anglia, Norwich Research Park, Norwich, NR4 7TJ England

**Keywords:** Dementia, Hip fracture, Inflammation, Cerebrospinal fluid

## Abstract

**Background:**

Hip fracture represents a substantial acute inflammatory trauma, which may constitute a significant insult to the degenerating brain. Research suggests that an injury of this kind can affect memory and thinking in the future but it is unclear whether, and how, inflammatory trauma injures the brain. The impact of Acute SystematiC inflammation upon cerebRospinal fluId and blood BiomarkErs of brain inflammation and injury in Dementia: a study in acute hip fracture patients (ASCRIBED) explores this relationship, to understand the effect of inflammation on the progression of dementia.

**Methods:**

This protocol describes a multi-centre sample collection observational study. The study utilises the unique opportunity provided by hip fracture operations undertaken via spinal anaesthesia to collect cerebrospinal fluid (CSF) and blood, to investigate the impact of acute brain inflammation caused by hip fracture on the exacerbation of dementia. We will recruit 200 hip fracture patients with a diagnosis or evidence of dementia; and 200 hip fracture patients without dementia. We will also recruit ‘Suitable informants’, individuals in regular contact with the patient, to provide further proxy evidence of a patient’s potential cognitive decline. We will compare these 400 samples with existing CSF and blood samples from a cohort of dementia patients who had not experienced a systemic inflammatory response due to injury. This will provide a comparison between patients with and without dementia who are suffering a systemic inflammatory response; with stable patients living with dementia.

**Discussion:**

We will test the hypothesis that hip fracture patients living with dementia show elevated markers of brain inflammation, as well as neuronal injury and Alzheimer-related plaque pathology, in comparison to (1) stable patients living with dementia and (2) hip fracture patients without dementia, as measured by biomarkers in CSF and blood. The findings will address the hypothesis that systemic inflammatory events can exacerbate underlying dementia and inform the search for new treatments targeting inflammation in dementia.

**Trial registration:**

ISRCTN43803769. Registered 11 May 2017.

## Background

Inflammation is a beneficial physiological response to tissue damage or infection. However, when inflammation is extensive or not fully resolved, this can damage healthy tissues and disrupt normal cellular function. Hip fracture represents a substantial systemic inflammatory trauma, common in older people, which may constitute a significant insult to the degenerating brain and therefore contribute to the progression or even the onset of dementia. Hip fracture in older people has therefore been linked with poor cognitive outcomes, including delirium in the short-term, increased dependency and cognitive decline, especially in patients with dementia [1–3].

The association and pathological role of inflammation in dementia has been extensively described [4]. Studies have shown that microglial cells (the brain’s main macrophage population) are activated in the vicinity of amyloid plaques in dementia [5]. More recent studies suggest that altered macrophage function may contribute to dementia [6]. Animal studies have shown that microglial activation is a consistent feature in dementia and there is evidence that inflammation contributes to the disease process [7, 8] but the physiological and molecular basis for this remains unclear.

Current evidence from human epidemiological studies, human data from blood, cerebrospinal fluid and imaging, and animal models, have established that alongside chronic localised inflammation resulting from and contributing to neurodegenerative diseases such as dementia, there is also neurodegeneration induced by acute inflammatory processes [9] and changes in amyloid processing [10]. Understanding this alternative route to neurodegeneration is becoming increasingly important as the population ages. This is because acute systemic inflammatory episodes, such as infection and inflammatory trauma, are common in older people with some evidence of this having both acute [11] and lasting [12] impacts on cognitive function. Therefore, it is plausible that such episodes are an important cause of decline in people living with dementia, which is clinically almost completely unaddressed.

With rapid advances in identifying and measuring/testing cerebrospinal fluid (CSF) and blood biomarkers of brain inflammation, brain injury and Alzheimer-associated amyloid β (Aβ) plaque pathology, there is the opportunity to study this in humans.

Previous studies in older people with acute systemic inflammation have been limited by small sample sizes, the lack of adequate control groups and, in particular, have not assessed the impact of inflammation on recently emerging biomarkers of new brain injury [13].

One of the other difficulties encountered by research in this area is that hip fracture is an emergency and studies cannot directly collect pre-fracture data. However, well-validated methods for the assessment of pre-fracture cognitive ability are available. The Informant Questionnaire for Cognitive Decline in the Elderly (IQCODE [14] is one example widely-used clinically and for research purposes.

In the United Kingdom (UK) a significant proportion of hip fracture patients undergo surgery within spinal anaesthesia [15]. This routine clinical procedure involves inserting a needle into the patient’s spinal space (subarachnoid space) and injecting anaesthetic into the CSF. In this way, CSF can be collected just before the initiation of anaesthesia, using the same needle that will be used to administer the anaesthetic agent. This means that older patients undergoing emergency hip fracture repair surgery are a suitable group in which to measure systemic inflammation, brain inflammation and CSF markers of brain injury.

The impact of Acute SystematiC inflammation upon cerebRospinal fluId and blood BiomarkErs of brain inflammation and injury in Dementia: a study in acute hip fracture patients (ASCRIBED) will use the opportunity provided by hip fracture operations undertaken via spinal anaesthesia to investigate the impact of acute systematic inflammation upon CSF and blood biomarkers of brain inflammation and neuronal injury and on the exacerbation of dementia. We will collect samples from patients with and without dementia who are suffering a systematic inflammatory response (the ASCRIBED cohort). We will compare ASCRIBED’s ‘unstable’ groups (termed as to refer to the inflammatory response) with an existing cohort of patients living with dementia who have not experienced a systemic inflammatory response from an injury (henceforth known as the ‘Oslo’ cohort). The Oslo cohort will therefore provide ‘stable’ comparators. The study will shed light on the ability of acute inflammatory trauma to produce new brain injury in a vulnerable older population. The findings will then inform the search for new treatments targeting inflammation in dementia.

## Methods

### Aims and objectives

In order to have specific measures informing on the severity of prevalent systemic inflammation at the time of lumbar puncture (i.e., the time of CSF collection), matched to those inflammatory mediators occurring in the CSF, we will quantify inflammatory mediators (including but not limited to IL-1β, TNF-α, IL-6) in both peripheral blood and in CSF. In order to assess brain injury we will measure CSF markers of brain injury (including but not limited to total and phosphorylated tau [T-tau and P-tau, respectively], neurofilament light [NfL] and neurogranin). Brain injury markers will also be measured in blood. Aβ42/40 ratio in CSF and plasma, measured using immunoassays (Meso Scale Discovery and Simoa methods, respectively), will be used as a biomarker of cerebral Aβ pathology. We will also collect an additional 2.5 ml of whole blood from patients. Several studies have recently been published using PAXgene blood collection tubes for later transcriptomic analysis. Our intention is to place ourselves in the position to examine blood signatures that associate with, and may be predictive of, particular CSF and clinical outcomes in our patients for later analysis. Banking these samples will enable further in-depth analysis and is in accordance with the trial ethical approval and consent process.

#### Primary objective

To determine whether hip fracture patients living with dementia show elevated markers of brain inflammation in comparison to (1) stable patients living with dementia and (2) hip fracture patients without dementia, as measured by biomarkers in CSF. CSF inflammation will be measured by TNF-α, IL-1RA, IL-1β, IL-6 and brain injury and biomarkers will be measured by NfL, neurogranin, T-tau, synaptotagmin and SNAP-25.

#### Secondary objectives

To determine whether the magnitude of the brain inflammatory response predicts the quantity of specific brain injury markers measured in the CSF. Magnitude of the brain injury will be assessed via brain injury markers T-tau, P-tau, NfL, neurogranin, synaptotagmin and SNAP-25 in CSF. We will also examine if patients who are Aβ-positive at baseline are more or less likely to have dementia or develop dementia at follow-up. We will also look for interactions of Aβ positivity with the other biomarkers in regards to clinical outcome.

### Design

**Fig. 1:**
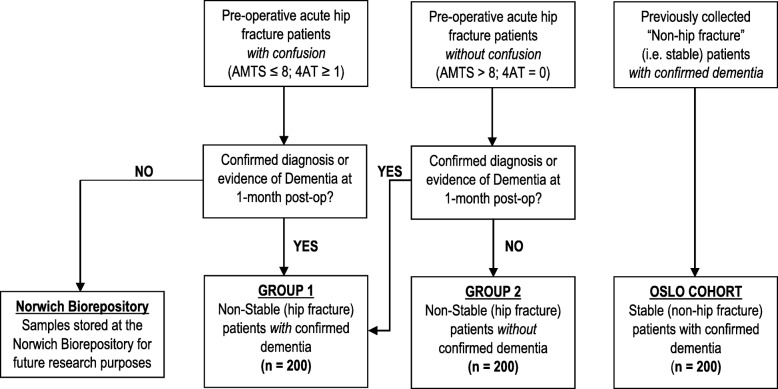
Study Diagram and Group Allocation of Patients

This observational study will recruit patients with proximal hip fractures who undergo surgery via spinal anaesthesia. The majority of patients admitted with a hip fracture are cognitively vulnerable. This may be a pre-fracture state or an acute reaction to the hip fracture. Clinically, patients arriving in acute settings do not always arrive with a confirmed dementia diagnosis. However, in the UK, it is routine clinical practice to cognitively screen hip fracture patients over the age of 60 years. In England, the Abbreviated Mental Test (AMT) is commonly used [16]. In Scotland, the 4AT is used as best standard practice [17]. Because evidence highlights that the mapping of a patient’s score on the 4AT on to the AMTS is possible [18, 19], we will use routinely available clinical data to pre-operatively assign recruited patients to one of two groups, either ‘confused’ or ‘non-confused’. In this way, we will employ the term confusion to reflect the real-world complexity of the acute hospital environment and initially assign patients accordingly, based on these existing cognitive clinical screening practices (see Fig. 1). Specifically, Group 1 patients will have a pre-op AMT score of ≤8 (England), or a 4AT score of > 1 (Scotland); and Group 2 patients will have a pre-op AMT score of > 9 (England) or 4AT score of 0 (Scotland).

Whilst these are indicative of the possibility that the patient may have some form of dementia/cognitive impairment, it is often not possible to obtain confirmative evidence until at least 1-month post-op. Using the AMT or 4AT scores allows us to allocate patients to a group at the recruitment stage. However, we will also take into account any subsequent evidence of dementia in the analysis, by gaining permission/consent to access a patient’s notes and/or where possible, a consented suitable informant (someone who has contact with the patient at least once a month face-to-face or via telephone) via the Informant Questionnaire for Cognitive Decline in the Elderly (IQCODE). This will inform what final group cohort the patient is then allocated to. In the absence of a formal documented diagnosis of dementia and in accordance with previous research [20, 21], patients with an associated suitable informant IQCODE score of 3.31 and above will be assessed as having sufficient evidence of dementia to be allocated to the corresponding group cohort.

### Setting

The study setting is acute trauma wards in hospitals across England and Scotland to which individuals suffering Neck of Femur (NoF) fractures are admitted. In all instances, the investigator(s) will be able to demonstrate a potential for recruiting the required number of suitable participants within the agreed recruitment period (i.e. the investigator(s) regularly treat(s) the target population). For these reasons, the study management team will target NHS Hospitals with large annual admission rates of NOF fractures with sufficiently high percentages of operations being undertaken via spinal anaesthesia. This information was readily available through the National Hip Fracture Database (NHFD) for all NHS Trusts in England [16]. In Scotland, we targeted large centres known to the study group and with existing expertise in collecting CSF for research purposes.

### Participants

We will be collecting samples and data from two groups of patient participants (*n* = 200 in each group). Due to the cognitively vulnerable nature of the patient population and feasibility learning in relation to dementia diagnosis rates of our target population [22], we will seek proxy information about pre-fracture cognition to inform grouping allocation for analysis. Consequently, we will also seek written consent from “suitable informants” as defined by the inclusion criteria below, to complete the Informant Questionnaire for Cognitive Decline in the Elderly (IQCODE). The recruitment of a suitable informant for each patient is desirable, but not essential. The eligibility criteria for patient participants and suitable informant participants are as follows:

Patient inclusion criteria:

Group 1: ‘Confused’ hip fracture patients

**Inclusion Criteria:**

1. Patient must have had a confirmed proximal hip fracture requiring an operation and be aged 60 or older at the time of operation;
2. Patient has a pre-operative Abbreviated Mental Test Score (AMTS) of 8 or below; or 4AT score of 1 or above;
3. Patient must be undergoing spinal anaesthesia.

**Exclusion criteria:**

1. Decision taken not to have hip surgery;
2. Patient has head trauma with bleeding as indicated by a CT scan;
3. Patient has confirmed diagnosis of Parkinson’s disease;
4. Patient not expected to survive beyond 4 weeks;
5. Patient’s fall and subsequent hip fracture caused by acute Stroke, indicated by CT and/or MRI scan and/or clinical examination;
6. Patient already enrolled in a Clinical Trial of an Investigational Medicinal Product (CTIMP).

Group 2: ‘Non-confused’ hip fracture patients

**Inclusion Criteria:**

1. Patient must have had a confirmed proximal hip fracture requiring an operation and be aged 60 or older at the time of operation;
2. Patient has a pre-operative AMTS of 9 or above; or 4AT score of 0;
3. Patient must be undergoing spinal anaesthesia.

**Exclusion criteria:**

1. Decision taken not to have hip surgery;
2. Patient has head trauma with bleeding as indicated by a CT scan.
3. Patient has confirmed diagnosis of Parkinson’s disease;
4. Patient not expected to survive beyond 4 weeks;
5. Patient’s fall and subsequent hip fracture caused by acute Stroke, indicated by CT and/or MRI scan and/or clinical examination;
6. Patient already enrolled in a Clinical Trial of an Investigational Medicinal Product (CTIMP).

### Suitable informants

**Inclusion Criteria:**

1. Individual has a minimum of once a month face-to-face or telephone contact with the patient;
2. Individual is able and consents to complete the IQCODE.

**Exclusion Criteria:**

1. Individual under 16 years of age.

### Recruitment and consent procedures

A three-phase recruitment process has been guided by conversations with clinical and academic collaborators and previous experience recruiting from this patient group [22].

1. Research Nurses will collaborate with relevant clinical staff (including but not exclusively the study ward Trauma Co-ordinators and key Emergency Department colleagues) to identify all new hip fracture admissions and screen for pre-recruitment eligibility;
2. Each patient (and where possible their potential suitable informant) will be approached by a Research Nurse who will provide information about the study as soon as clinically appropriate. During this initial approach, the Research Nurse will also assess the mental capacity of the patient;
3. The Research Nurse will approach the patient (where possible) and the identified suitable informant to obtain full written informed consent. In cases where written consent is not possible, ethical approval allows for witnessed verbal consent.

In English trial sites, in line with Principle 1 of the Mental Capacity Act 2005 [23], a potential patient participant will be assumed to have capacity until it is established otherwise. When this is the case and all practical steps to help them to engage in the decision making process have been tried (Principle 2 of Mental Capacity Act 2005), the site trial team will seek a personal consultee. This person will be someone who is engaged in care for the participant (not professionally or for payment) or is interested in his/her welfare and is prepared to be consulted. This may be a family member, carer or close friend, or attorney acting under Lasting Power of Attorney. This person can also act as a suitable informant if they fulfil the inclusion criteria.

If a potential personal consultee is not available or declines to take part, alternatively a nominated consultee will be sought. This will be a person independent of the research study and who is willing to be consulted about the participation of a person who lacks capacity where reasonable steps have been taken to identify a personal consultee. This may be someone who knows the patient in a professional capacity e.g. social worker, ward staff member, paid carer or GP, provided they have no connection to the research study.

In Scottish sites, in line with the Adults with Incapacity Act 2000 [24], where a potential patient participant is assessed not to have capacity, a welfare guardian, welfare attorney or nearest relative will be sought and asked to consent in relation to participation in research (this person will be henceforth known as a legal representative). This procedure will be undertaken once an assessment of capacity has been made in relation to the specific decision regarding the research participation, any barriers to participating in the consent process have been removed and the local research worker feels the individual cannot retain information long enough to use it in order to arrive at a decision.

Legal representatives may be involved in conversations regarding the consenting process. However, they will be asked to differentiate between expressions of their own views and reporting the known values and/or views of the potential patient participant. If the potential participant is unable to consent for himself or herself, then consent will be sought on their behalf from a suitable legal representative.

In cases where gaining full written consent is not possible research workers may take witnessed verbal consent (patients or legal representatives) or agreement (personal consultees). For patients this may be needed due to an inability to write because of injury. With personal consultees or legal representatives this may be due to distance therefore study information may be conveyed over the phone with relevant forms sent via email if appropriate. Where witnessed verbal agreement/consent is taken, full written agreement/consent will be sought where practically possible. A record of all witnessed verbal consent will be added to the patient’s notes.

In both England and Scotland, if during a follow-up assessment the patient is assessed by a local research worker to have regained capacity (a possibility in the case of some cognitive impairments such as delirium); he/she will be approached about continuing to participate in the study and asked to give informed consent. Should they choose to withdraw from the study at this point, the study team reserve the right to retain any data and samples collected up until the point of the patient’s withdrawal. This will be clearly stated in the patient and Consultee (English sites)/Legal Representative (Scottish Sites) Information Sheets.

This three-phase process will be closely monitored to identify trends that might be leading to over or under recruitment from specific groups. For example, if sites are consenting purely via personal consultees (England) or legal representatives (Scotland), monitoring will enable corrective actions and provide information to mitigate these recruitment trends.

### Recruiting patients with fluctuating and/or reduced capacity in England and Scotland

The aims of this study are incompatible with only enrolling patients with minimal or mild confusion. It is important to ensure findings are broadly applicable to those patients with a pre-existing diagnosis or evidence of dementia. Participants who lack capacity to give informed consent must therefore be included. In this situation, the patient’s agreement to participate will still be obtained to their best level of understanding (in line with legislative frameworks in England [23] and Scotland [24]). Where patients in England are assessed as lacking capacity to make a decision regarding their initial or continued involvement with the study, we will seek a personal or nominated consultee agreements [25]. In Scottish study sites where a patient is assessed not to have capacity, a legal representative will be sought and asked to consent in relation to the patient’s participation in the research [26].

### Approaching patients post-operatively

Where possible, the patient will be approached at a clinically suitable time approximately 48 h (± 4 h) following their operation. However, in order to facilitate patient recruitment and because successful collection of a sufficient number of pre-operative CSF samples is the priority for this study, sites are encouraged to screen and recruit patients from Monday-Friday. This is on the understanding that should a patient be consented on a Thursday/Friday, it may not be possible to complete the 48-h follow-up due to insufficient Research Nurse cover during weekends.

**Fig. 2:**
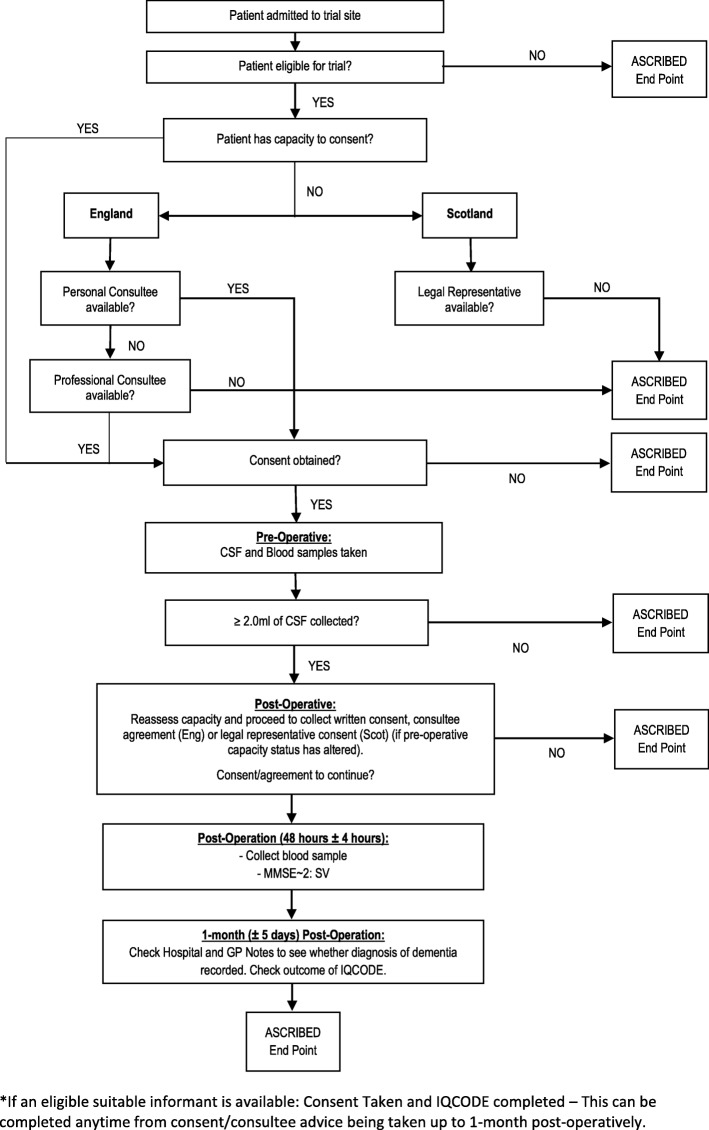
Recruitment Overview and Participant Flow

During the 48-h follow-up point, we will aim to collect the post-operative blood sample and Mini-Mental State Examination - 2nd Edition, Short-Form version (MMSE~ 2: SV) data. As appropriate, the research nurse will remind the patient of the study, reassess capacity (as required) and complete pre-consented study related procedures. In English sites, for patients who previously provided informed consent on their own behalf but are as assessed as having since lost capacity at this follow-up point, we will seek a personal or nominated consultee agreements [23]. As part of the patient consent form for Trusts based in England, patients will be asked to provide contact details for someone who may be willing to act as a personal consultee in the event that the patient loses capacity. Patients will also be asked to sign an advanced statement of intent, stating that should they be assessed as having lost capacity post-operatively, they would still like to be involved in the study should a consultee be available. A more detailed overview of the recruitment process is shown in Fig. 2: Recruitment overview.

### Data collection (Table 1)

**Table 1:** Sample and data collection schedule

Timepoint	Admission/Pre-Op Period	Day of Operation	Post-Op Period	
		Day 0	48 (± 4 hours) post-op	Time 1 (1 month ± 5 days)
Consent/ Agreement	X		X^a^	
AMT and/or 4AT	X		X^b^	
Collection of blood EDTA sample (6 ml)		X		
Collection of blood serum clotted sample (10 ml)		X	X^b^	
Collection of Cerebrospinal fluid (CSF) sample (≥ 2.0 ml)		X		
MMSE~ 2: SV			X^b^	
IQCODE *(To be completed by the suitable informant)*			X^c^	
Evidence of dementia from patient’s medical/GP records				X^c^
Medication information				X^c^
Collection of blood PAXgene RNA sample (2.5 ml)		X		

^a^Taken if patient’s capacity status has changed from pre-operative time period (Eng. only);

^b^Should the time window be unworkable, research nurses will collect MMSE~ 2: SV data and bloods at the next earliest opportunity but not beyond 60 h post-op;

^c^Can be gained at any point before the 1-month (± 5 days) time period elapses

#### Day of operation

The research nurse and/or anaesthetist at the time of the hip fracture operation will be responsible for collecting 18.5 ml of whole blood (1 × 6.0 ml of blood ethylenediaminetetraacetic acid (EDTA) tube, 2 × 5.0 ml serum tube and 1 × 2.5 ml PAXgene tube) and the collection of 2.0–6.0 ml of CSF (anaesthetist only).

Sites will be instructed to centrifuge CSF samples within 1 h of sample collection at 2000G for 10 min. If CSF samples are not centrifuged within a maximum of 2 h of collection, site teams will be informed to destroy the samples and alert the study management team accordingly. Blood serum samples will be centrifuged at 2000G for 15 min, within 1 h of collection. If any blood serum samples are not centrifuged within 3 h of collection, they must be rejected and destroyed; and the study management team notified accordingly. The EDTA and PAXgene samples will not require centrifugation and sites are instructed to leave these to rest at room temperature for 2 h, following inversion.

Once processed and ready for storage, the CSF, EDTA and blood serum samples will be aliquotted into 0.5 ml samples within 1.5 ml capacity Cryotubes, and stored in a specific patient Cryobox. Both the Cryobox and all of the individual Cryotubes used for patient sample storage are labelled with the patient’s unique study identifier number and colour coded to match the sample type being stored. PAXgene samples are also labelled accordingly but remain stored in their initial vacutainers, inside the corresponding patient’s Cryobox. The Cryobox will then be stored in a -80° Celsius freezer at the local research site. All of the sample collection, processing and storage times for each patient will be recorded within the study’s electronic database for monitoring purposes.

Once a site has successfully recruited and collected samples for 10 patients, the study management team will arrange for a courier to collect and deposit the samples at the Norwich Biorepository for long-term storage, until the final sample analysis is ready to be started. All sample transfers will be completed on a same day delivery basis, using dry ice to maintain sample cooling. Once at the Norwich Biorepository, samples will be monitored against the electronic database records and sample transfer log, for completeness and accuracy. Samples will then be deposited in a -80° Celsius freezer within the Norwich Biorepository. Any discrepancies will be followed up with the local research team and recorded in the electronic database accordingly.

Spinal anaesthesia will be performed according to local trust procedures. After placement of the needle to deliver the spinal anaesthetic, and prior to administration of the anaesthetic agent, a sample of between 2 and 6 ml of CSF will be collected. Patients unable to provide a sufficient CSF sample will be withdrawn from the study and any prior samples collected destroyed according to Local Trust Policy.

During collection of the CSF, the patient will be monitored. Should the patient’s discomfort become too great, the anaesthetist will stop collecting the CSF. Headaches (‘post-dural-puncture headache’ or ‘PDPH’) are a common side effect of spinal anaesthesia and typically occur within two to 3 days following the procedure. After taking advice from anaesthetists, it was identified that the risk of patients experiencing a PDPH may be slightly higher for patients taking part in this study because of the additional CSF withdrawn. The incidence of PDPH’s will therefore be monitored as part of routine care using standard local procedures. Any PDPH observed by the clinical team will be assessed for severity and reported as an adverse event. The incidence rates of PDPH’s will be monitored and review by the Data Monitoring and Ethics Committee (DMEC) and Study Steering Committee (SSC). However, the risk is expected to be negligible.

#### Post-op 48 h (± 4 h)

2 × 5.0 ml blood (serum tube) will be collected from the patient. Every effort will be made to collect post-operative bloods within this time window, but this may not always be possible. Therefore, research nurses will collect the MMSE~ 2: SV data and bloods at the next earliest opportunity but not beyond 60 h post-op. The time point at which these samples and data are collected will be noted and fed into the analysis. Sites consistently collecting samples outside the 48 (± 4 h) window will be reviewed by the Study Management Group (SMG) who will decide if they should be withdrawn. For patients recruited on a Thursday or Friday, it is accepted that this follow-up may not be possible due to insufficient Research Nurses across weekends.

#### Post-op 1-month (± 5 days)

The 1-month post-op period will provide clinical teams with an opportunity to contact the patient’s General Practitioner (GP) and review their case notes to assess if the patient has a pre-existing documented diagnosis of dementia, as well as record some additional clinical measures and test results. If the patient has an eligible suitable informant, clinical teams will also use this time to complete IQCODE assessment if they have not already done so.

### Sample size

We will recruit 200 patients with dementia and hip fracture; and 200 patients without dementia but who have experienced a hip fracture. This sample size is pragmatically based upon what would appear to be achievable in the time available and with consideration of likely statistical power. Without adjustment, a sample size of 200 subjects per group will provide statistical power of 90% to detect a mean between group mean differences of 0.33 standard deviations in any outcome variable using a two-sided significance level of 5%. Assuming confounding variables entered into a General Linear Model ‘explain’ no more than 25% of the total variation (i.e. the co-efficient of determination, R2, is less than 0.25), then this sample size should provide 90%. Power to detect an ‘adjusted’ mean difference of around 0.37 residual standard deviations [27]. In either case, this would be deemed a relatively small effect to be detected with high probability.

Data will be collected initially from two different groups:

- Group 1: Pre-operative acute hip fracture patients with confusion;
- Group 2: Pre-operative acute hip fracture patients without confusion.

In respect of Group 1 (those with confusion), the AMT (England) and 4AT (Scotland) score indicate that a patient may be living with dementia. However, this may not be confirmed until 1-month post-op when reviewing the patient’s case notes, contacting their GP or reviewing their relevant Suitable Informant’s IQCODE Scores. Based on prior research, we anticipate that up to 50% of patients who have an AMT score of 8 or less (England) or 4AT score of 1 or above (Scotland) will have dementia (diagnosed or undiagnosed/vectored) [22]. Therefore, up to 400 may need to be recruited to this group. Recruitment will be monitored and stopped for Group 1 as soon as we receive 200 patients with confirmed dementia required for the study.

We will also collect data from patients without confusion (Group 2), who are unlikely to be confirmed with dementia at 1-month post-op. These patients will be included in the non-dementia group. Again, recruitment will be monitored and stopped from this group once 200 non-dementia patients have been included. The number required from this group will be dependent upon the non-dementia confirmation rate for this group.

There will be a number of patients from Group 1 (Pre-operative acute hip fracture patients with confusion) for whom we cannot find evidence of dementia at 1-month post-op. The samples and data from this (confused, non-dementia) group will be deposited into a biobank at the Norwich Biorepository, for use in future research studies. In cases where patients were initially in Group 2 (Pre-operative acute hip fracture patients without confusion) but where evidence of dementia is available at 1 month post-op, we will reallocate these patients to the dementia patient group.

Comparable data will also be provided from a third group (Oslo Cohort) of 200 ‘stable’ patients living with a confirmed dementia diagnosis, taken from existing memory clinic data (Norwegian Registry of Persons with Cognitive Symptoms (NorCog) (Reference: S-08143a and 2017/371). Samples for this group are already available, as lumbar puncture is part of the diagnostic workup of patients included in the Norcog registry (Reference: S-08143a). These samples were analysed in 2017 at Sahlgrenska for the following: Aβ38, Aβ40, Aβ42, 10xAb42/Ab40, YKL-40, IL-1β, IL-6, IL-8, TNF-α, G36-NG2. The respective regional committee responsible have already provided permission to compare these results with those gathered in the present study.

Thus, we shall assemble data from 3 groups (hip fracture and dementia, hip fracture and non-dementia, stable and dementia), each with an expected 200 subjects. Please (see Fig. 1: Study diagram and group allocation of patients).

### Analysis

All analyses will be conducted according to a detailed Statistical Analysis Plan (SAP), agreed by the Study Management Group (SMG) prior to analysis. A summary of the main analyses are given below however:

#### Primary hypothesis

We will address the primary hypothesis, that systemic inflammation arising from hip fracture leads to an acute brain injury, by comparing the level of inflammatory and neuronal injury CSF and blood markers between the three groups defined above. Accordingly, we predict raised inflammatory and injury markers for the confirmed dementia-hip fracture group compared to the medically stable dementia group (Oslo cohort) and compared to the non-dementia hip fracture group.

Each of the markers will be compared across groups using a general linear model with the marker as the dependent variable (i.e. a separate model for each biomarker). The initial model will simply include group as an explanatory factor. A further model will then be constructed, including potential confounding variables, such as age, to provide an adjusted between group mean difference (comparing fracture patients with dementia to fracture patients without and fracture patients with dementia to stable dementia patients), together with 95% confidence intervals and significance test. In the event of the residuals for these models not appearing normally distributed, an appropriate transformation will be applied, such as a logarithmic transformation. We also predict that patients with dementia will have significantly worse cognitive and functional informant-based scores. A similar analysis will be conducted with cognitive and functional scores as the dependent variable.

#### Secondary hypothesis

The secondary hypothesis is that the magnitude of the brain inflammatory response will predict the quantity of specific brain injury markers (phospho-tau, NfL, neurogranin, synaptotagmin, SNAP-25) measured in the CSF. The strength of inter-relationship between the inflammatory and injury markers outlined will be examined using correlation coefficients. These will also be adjusted for potential confounding factors using partial correlation coefficients.

Analysis of the samples will take place at UEA, Trinity College Dublin and the University of Gothenburg. Should additional information become available during the course of the study, we will ensure that we use the most appropriate analysis available to answer the research questions.

- CSF will be analysed for a number of inflammatory and neuronal injury markers. These include, but are not limited to: TNF-α, IL-1RA, IL-1β, IL-6, sTREM2, YKL-40, T-tau, P-tau, Aβ38, Aβ40, Aβ42, neurogranin, synaptotagmin and SNAP-25;
- Blood collected pre-operatively and at 48 h (± 4 h) will be analysed for TNF-α, IL 1RA, IL-1β, IL-6, T-tau and NfL;
- Blood collected pre-operatively will also be genotyped for the APOE ε2/ε3/ε4 polymorphism at UEA;
- PAXgene blood for later transcriptomic analysis looking for blood signatures that associate with, and may be predictive of, particular CSF and clinical outcomes in our patients.

## Discussion

Despite significant investment, disease-modifying treatments for dementia are still absent and there has been no significant treatment breakthrough for 15–20 years [28]. Inflammation is a vital part of the immune system’s response to injury and infection which may become harmful if exaggerated or unresolved. There is now growing evidence that harmful inflammation in the brain is aetiological and contributed to the pathophysiology of dementia [29].

Recent research highlights acute illnesses or injuries that cause inflammation throughout the body, such as infection, trauma and surgery, can accelerate the speed of decline in dementia [30, 31]. For example, an infection in a hospitalised older person with dementia is linked to a higher long-term worsening of that person’s symptoms. The underlying mechanisms linking inflammation, cognition and dementia progression remain greatly under-researched, with almost no studies in humans. This lack of research impacts on the search for new treatments targeting inflammation in dementia.

Thus this study will develop understandings of the role of inflammatory response in dementia and support developing pharmaceutical interventions. Additionally it will inform ways to predict deterioration in dementia. Exploration of new potential disease pathways remains essential for finding new therapeutic targets.

## Data Availability

Not applicable.

## Abbreviations

AMTS: Abbreviated Mental Test Score
ARUK: Alzheimer’s Research UK
BTP: Best Practice Tariff
CSF: Cerebrospinal fluid
DMEC: Data Monitoring and Ethics Committee
EDTA: Ethylenediaminetetraacetic acid
GP: General Practitioner
IQCODE: Informant Questionnaire for Cognitive Decline in the Elderly
MMSE~ 2: SV: Mini-Mental State Examination - 2nd Edition, Short-Form version
NHFD: National Hip Fracture Database
NHS: National Health Service
NOF: Neck of femur
PDPH: Post-dural puncture headache
SAP: Statistical Analysis Plan
SMG: Study Management Group
SSC: Study Steering Committee
UK: United Kingdom
